# Tick-borne encephalitis virus associated with foetal death in a bitch, a case report

**DOI:** 10.1186/s12985-025-02965-7

**Published:** 2025-10-13

**Authors:** Bodil Ström Holst, Anna Bonnevie, Jennifer Spens, Johanna F. Lindahl, Anna Huupponen, Pernilla Syrjä, Anne-Lie Blomström

**Affiliations:** 1 Department of Clinical Sciences, Faculty of Veterinary Medicine and Animal Science, Swedish University of Agricultural Sciences, Uppsala, Sweden; 2 Swedish Veterinary Agency (SVA), Uppsala, Sweden; 3 Department of Medical Biochemistry and Microbiology, Uppsala University, Uppsala, Sweden; 4 Department of Veterinary Biosciences, Faculty of Veterinary Medicine, University of Helsinki, Helsinki, Finland; 5 Department of Animal Biosciences, Faculty of Veterinary Medicine and Animal Science, Swedish University of Agricultural Sciences, Uppsala, Sweden

**Keywords:** Foetal death, Tick borne encephalitis virus, Flavivirus, Canine, Foetal resorption

## Abstract

**Background:**

For the first time, a case of vertical transmission of TBEV in a dog associated with foetal death is described.

**Case presentation:**

A six-year-old beagle bitch experienced foetal death from day 49 in pregnancy. A caesarean section was performed on day 56, and one live and three dead pups in different stages of resorption were delivered. Black mucoid, non-smelling foetal membranes surrounded the dead foetuses. The live-born foetus died despite efforts to save it and was sent for autopsy together with the placenta. Autopsy demonstrated lung atelectasis and no malformations. A mild acute necrotizing placentitis was diagnosed on histopathology. Selective bacteriological cultures for *Brucella canis* from blood, vagina and the foetus were all negative, as was PCR for canine herpes virus (CHV). Viral metagenomics analysis identified the presence of tick-borne encephalitis virus (TBEV) in the placental tissue and in situ hybridization revealed TBEV in the trophoblasts. The bitch had antibodies to TBEV. One year later, the bitch had a normal pregnancy and whelping.

**Conclusion:**

With the spread of both ticks and TBEV, infection with TBEV should be given further consideration as a potential differential diagnosis in cases of foetal death in dogs.

## Background

Prenatal and neonatal deaths hamper strategic breeding and constitute a welfare risk for the bitch. To enable correct preventive measures to be undertaken, it is therefore important for the dog breeder and the veterinary community to identify their causes. Prenatal death is common in dogs, with resorption of embryos being present in as many as 43–48% of pregnancies [1, 2]. In late pregnancy, foetal death may be followed by retention and mummification of the foetus, or by abortion. Neither mummification nor abortion are commonly diagnosed. Neonatal mortality is reported to range between five and 35% but may in individual breeds be higher [3–5].

Prenatal and neonatal death may be categorized as having either non-infectious or infectious causes. There is a range of non-infectious causes to pregnancy loss in the bitch, including uterine pathologies and genetic defects. Infections are common causes of death in neonatal pups, and bacterial infections are attributed to more than half of the deaths in this group [6]. *Brucella canis* is an important infectious agent with trophism for reproductive tissue, causing reproductive disturbances in both male and female dogs [7]. In female dogs, *B. canis* has often been associated with abortions in late pregnancy and with stillborn and weak pups [7]. *B. canis* is not endemic in Sweden but emerging in Europe [8], and clinical infections in Western Europe are commonly associated with imported dogs.

Besides *B. canis*, which is a primary pathogen of the reproductive tract, occasional reports have been published on abortion in dogs caused by other infectious agents, including salmonellosis [9], leptospirosis [10], campylobacteriosis [11, 12], listeriosis [13] and viruses such as canine minute virus [14]. In other animals and humans flaviviruses are important causes of disease, and many flaviviruses have been described to be transmitted vertically, with subsequent infection of the foetus. One such flavivirus is Japanese encephalitis virus (JEV), which primarily causes encephalitis in humans. In pigs, JEV causes reproductive disease with stillbirth, mummification, and foetal death when immunologically naïve gilts are infected during the middle trimester [15–17]. JEV is not endemic in Sweden, in contrast to another flavivirus, tick-borne encephalitis virus (TBEV). In endemic areas, infections with TBEV are often subclinical in dogs [18], but they are also an important infectious cause of neurological disease [19–22]. Intrauterine transmission has been suggested as the cause of fulminant TBEV infection with neurological signs in a litter of pups [23].

An etiological diagnosis of canine abortion is hampered by the fact that it is not uncommon for the bitch to ingest aborted foetuses, making autopsies and sampling for infectious agents impossible. When present, the foetuses are often partly or fully autolysed, complicating analyses. The cost of autopsies and additional analyses deter many dog breeders from proceeding with investigations. Consequently, knowledge of both the scale of the problem with canine prenatal death and its causes remain limited.

## Case presentation

A six-year-old beagle bitch, 17.1 kg, was presented to the veterinary teaching hospital at the Swedish University of Agricultural Sciences (SLU) on day 49 after the last mating because of polyuria (PU), polydipsia (PD), and suspected foetal resorption. The bitch had previously been examined by a veterinarian at a local clinic because of PU/PD and an enlarged abdomen from day 32 after mating, and four foetuses, plus possibly one partially resorbed, had been identified. The bitch had been imported from Russia, and had had two previous litters after normal parturitions, the last one 2½ years ago. The sire of the present pregnancy had also been imported from Russia. The bitch had mated the same male one year ago, without conceiving. Progesterone analyses to determine the optimal day for mating had neither been performed at that time nor at the present pregnancy.

The bitch presented with a heart rate of 100 bpm and a slightly depressed general condition. Pregnancy diabetes was excluded based on normoglycemia and a normal serum fructosamine concentration. Ultrasound of the reproductive tract revealed one dead foetus, one foetus in distress (heart rate < 180 bpm), and two foetuses without signs of foetal stress (heart rates > 220 bpm). Four incompletely mineralized foetuses were visible on radiology; no teeth or mineralization of phalanges were seen. No treatment was initiated, but a follow-up visit was scheduled one week later, day 56 after the last mating.

At day 56, the bitch did not have PU/PD but had been inappetent for two days. Her general condition was good, and she was bright and alert. A blood sample revealed a normal concentration of C-reactive protein (< 7 mg/L). Progesterone was analysed with Immulite (Siemens), and the concentration was 7.1 nmol/L. Ultrasound revealed one foetus with normal cardiac activity (225 bpm) and two dead foetuses. Radiography visualized three fully mineralized foetuses and the vertebral column of a fourth foetus, but no intrauterine gas or skull step formations.

**Fig. 1:**
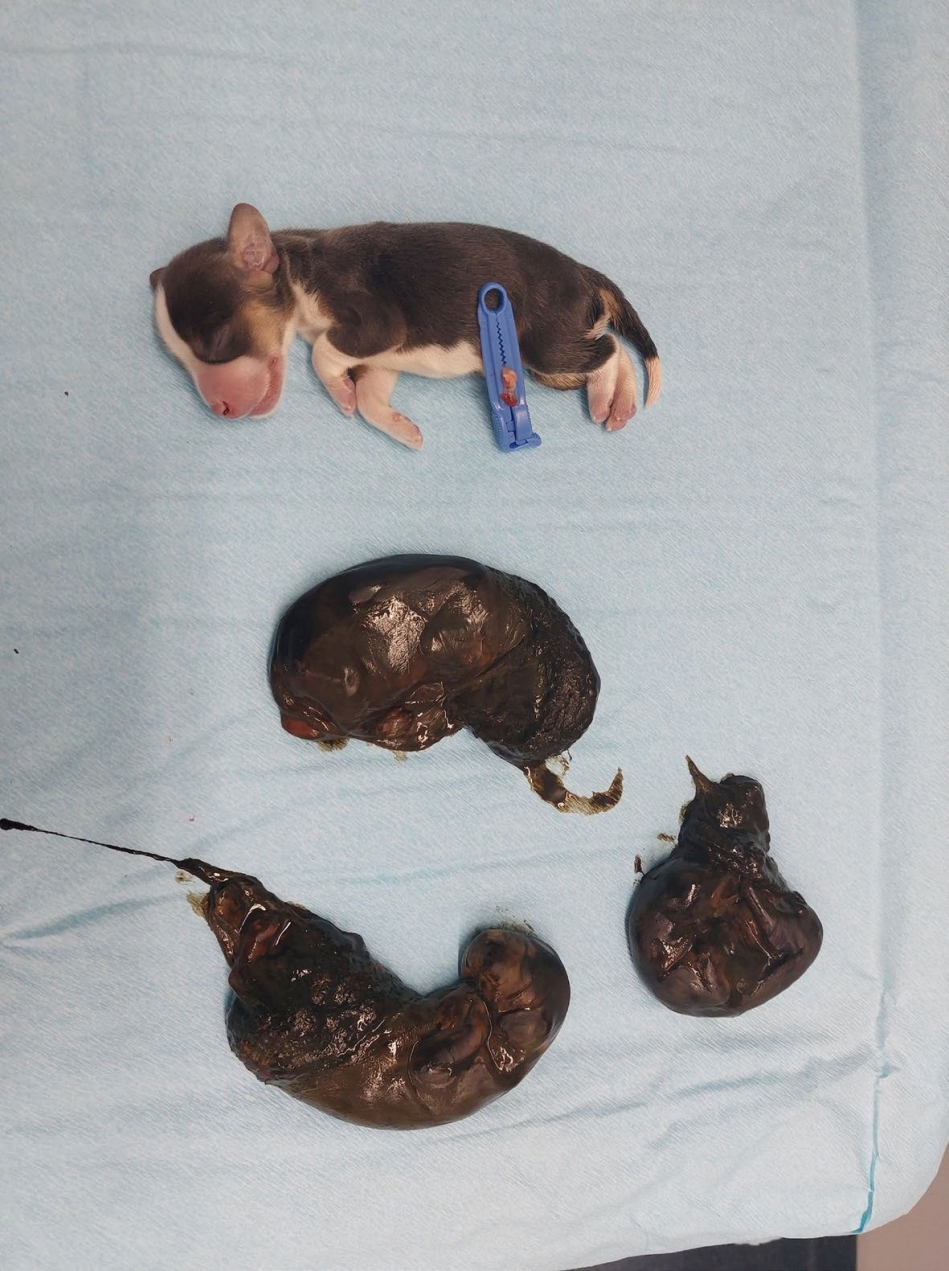
At the caesarean section, one live and three dead foetuses in different degrees of resorption were delivered

To save the last pup, a caesarean section was performed. One live and three dead pups were delivered. The dead foetuses were in different stages of resorption (Fig. 1). Black mucoid, non-smelling foetal membranes surrounded the dead foetuses. There was no pus, and the uterus appeared normal. The heart rate of the live pup was low, 60 bpm, and the attempt to save it was unsuccessful. Although the clinical picture was not typical for a bacteriological infection, foetal death in late pregnancy in a bitch that had been imported from a country where *B. canis* is endemic [24] motivated targeted sampling for *B. canis*.

Samples for blood culture and serology and a vaginal swab for bacteriological culture for *B. canis* were collected from the bitch. The live-born foetus with placenta was sent for autopsy, bacteriological culture for *B. canis* and PCR for canine herpes virus (CHV). Samples from the foetus (liver, lung, kidney) and placental tissue were also analysed using viral metagenomics. The most important differential diagnosis was *B. canis*, considering the clinical signs in an imported bitch. Other bacteria such as *Leptospira*, *Campylobacter* or *Listeria*, and viruses such as CHV, parvovirus or a flavivirus, were also considered. Salmonellosis was considered a less likely differential because abortions are commonly associated with systemic disease in the bitch [9].

**Fig. 2:**
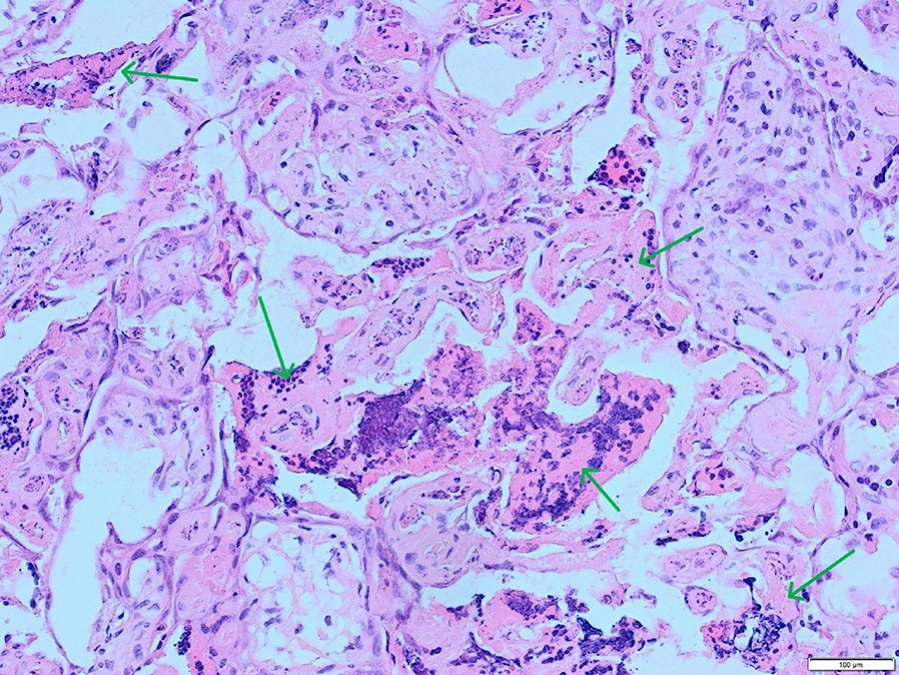
Necrotic foci in the placenta, with degeneration of tissue with mineralisation (green arrows). HE, 20x

### Autopsy

An autopsy was performed on the pup and a range of tissue samples (liver, kidneys, spleen, lungs) were collected and fixed in neutral-buffered 10% formalin, prepared and stained with routine stain: hematoxylin and eosin (HE) staining.

Macroscopically, the lungs were dark and sank in fluid, consistent with complete lung atelectasis, indicating that the pup had not breathed. There were no macroscopic or microscopic lesions in the liver, kidneys, spleen or lungs.

**Fig. 3:**
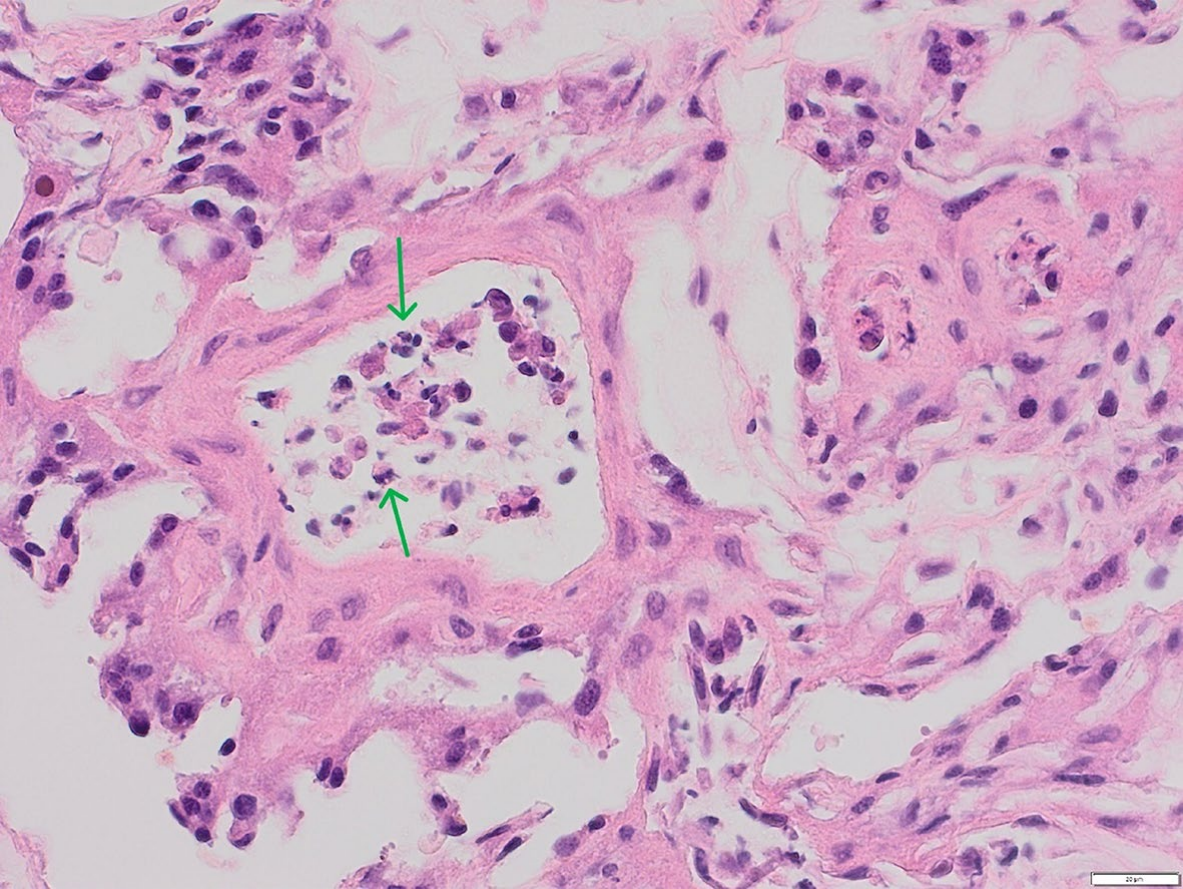
Blood vessel containing degenerating leukocytes, mainly neutrophils (green arrows), located close to a necrotic foci (not included in this field). HE, 40x

**Fig. 4:**
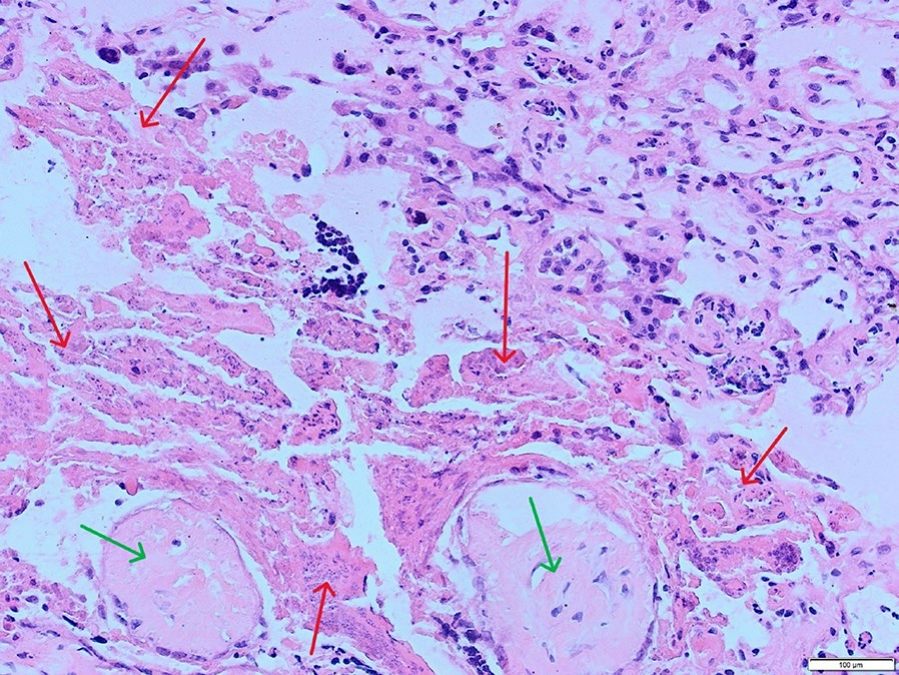
Necrotic foci (red arrows) in the placenta, with intravascular trombi (green arrows). HE, 20x

No macroscopic lesions were present in the placenta. Histopathological examination demonstrated features consistent with mild, multifocal, acute necrotizing placentitis. Necrotic areas were present multifocally in the placental tissue, extending from the junctional zone into deep areas of the labyrinth zone. Moderate amounts of scattered dystrophic mineralization and areas of eosinophilic cells with loss of distinguishable cell borders, as well as degenerated fragmented nuclei, were present. Surrounding cells were degenerated with atypical morphology, but in some areas, there was a mild presence of neutrophilic granulocytes (Fig. 2). These were located in blood vessels and grouped in the placental tissue (Fig. 3). There was a diffuse presence of amorphous basophilic material, compatible with mineralization and cell debris. Small, dark brown-black pigment granules were accumulated close to areas of necrosis and to the surface. There was a mild degree of bacterial presence, both cocci and rod-shaped bacteria, mostly affecting the surface and outer parts of the placenta. Multifocally in the tissue, there were blood vessels containing eosinophilic fibrinous material, consistent with microthrombi (Fig. 4). Infection with *Leptospira spp*., *Campylobacter spp*. or *Listeria spp*. were considered less likely based on histopathology.

Gram staining confirmed a mild presence of bacteria in the outer parts of the placenta, cocci and rods of both positive and negative variety, but none in the areas of necrosis. Van Kossa staining confirmed a clear presence of mineralization but also stain negative areas with non-mineralized debris. Perls Prussian blue staining indicative of the presence of hemosiderin was positive in outer pars of the placenta. This demonstrated that the dark pigment granules seen in the tissue were to some degree remnants from old haemorrhages. Period acid shift staining did not indicate any visible signs of damage on the basal lamina of the blood vessels.

### Bacteriological culturing, serology for B. canis and PCR for CHV

Selective bacteriological cultures for *B. canis* were performed according to standardized protocols based on Standard Operating Procedure from the European Union Reference Laboratory for Brucellosis. Culture samples from blood and vagina of the bitch and from a pool of liver, lung, kidneys and spleen of the foetus were all negative. Serology was positive with a titre of 1:180 (cut-off 1:100) using an ELISA (EVL, Holland) and with at titre of 100 UI/mL (cut-off 50 UI/mL) using a complement fixation test, but negative using a microplate agglutination test (ANSES, France) and using lateral flow immunoassay (Kit C. Brucella Ab Antigen, BioNote). The ELISA was repeated three times, with three months between the first and the last sampling, with negative results (titres < 1:100) in all runs. Based on the negative bacterial cultures and the negative results in the follow-up serology, the suspicion of infection with *B. canis* was excluded. The PCR for CHV was a modification of [25], exchanging the AgPath ID One-step RT-PCR kit with the KiCqStart Probe RT-qPCR ReadyMix. The result was negative.

**Fig. 5:**
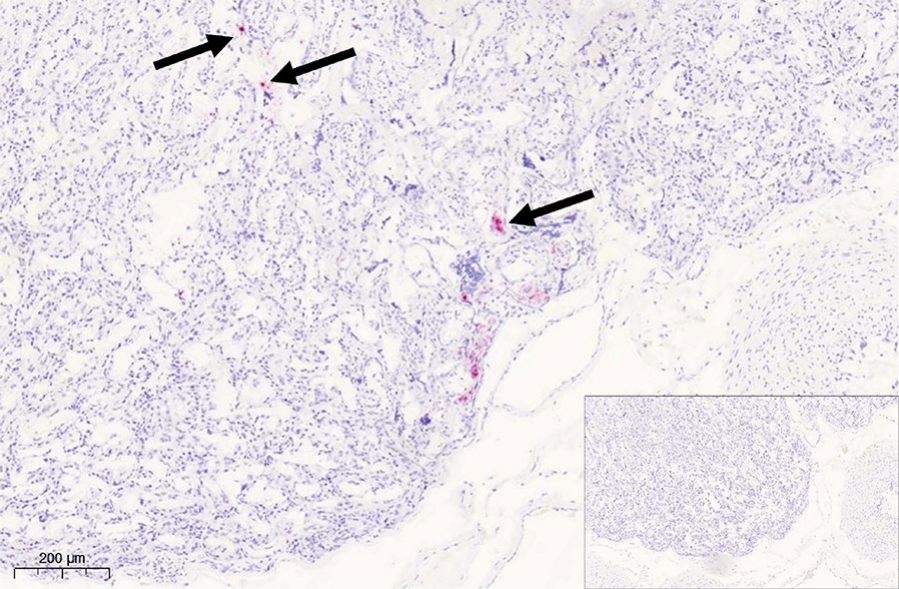
Canine placenta: a multifocal positive hybridization signal is seen in the deep parts of the placental labyrinth zone (arrows). Inset: negative ISH control section processed without probe. RNA Scope ISH for TBEV

### Viral metagenomics

When infection with *B. canis* and CHV had been excluded, placental tissue and internal organs (liver, lung, kidney) from the foetus were analysed using viral metagenomics, as previously described [26]. In short, the samples were homogenised using bead-beating (ck14; precellys), filtrated (0.45 μm), and the supernatant was collected after centrifugation. DNA and RNA were extracted separately using GenJET genomic DNA purification kit and Trizol/GenJET RNA purification kit. Prior to the DNA extraction, the supernatant was treated with DNase (10U DNase turbo) and RNase (2 µg RNase A). The RNA was on-column treated with DNase (QIAGEN RNase-Free DNase) during the RNA extraction and, in addition, rRNA depletion (Illumina Ribo-Zero plus) was carried out after the RNA extraction. The extracted RNA and DNA were randomly amplified using single Sequence-Independent, Single-Primer-Amplification (SISPA). Prior to Nanopore sequencing (Ligation Sequencing Kit (SQK-LSK109)), the SISPA products from the different tissues were pooled into one sequencing sample. The Nanopore sequencing yielded around 10 million reads (BioProject ID: PRJNA1267069) and CLC genomic workbench (v24) was used to remove low-quality reads and perform *de novo* assembly. Most of the reads were assembled into 155 902 contigs, while 362 823 reads remained unassembled. The assembled contigs and the remaining unassembled reads were annotated using Diamond (blastx) [27] and Megan7 [28]. Out of the analysed contigs/unassembled reads, 210 were classified as viral. Most of these reads were classified as retroviruses or as phages. However, interestingly, sequences classified as tick-borne encephalitis virus (TBEV) were also identified in the data set. A reference assembly towards a TBEV genome (MG589938) demonstrated that although the complete genome could not be obtained, reads were dispersed across the TBEV genome. Based on these sequences, primers were designed and with PCR TBEV virus could be confirmed in the placental tissue, but not in liver, kidney or lung tissue. Sanger sequencing (Macrogen Europe) of the amplified products additionally confirmed the correct amplification and showed that the identified TBEV had high (99 − 97%) nucleotide sequence similarity to different European TBEV isolates across the analysed genome regions.

### Serology

After detection of TBEV in the bitch, a serum sample, collected at the time of the caesarean section, was screened with a commercial competitive ELISA kit for detection of IgG antibodies against flaviviruses (IDvet company Grabels, France), and was found to be positive. This commercial kit uses microwells precoated with the pr-E protein of West Nile Virus (WNV) and detects both antibodies against WNV and cross-reacting antibodies induced by other flaviviruses [29, 30]. Tick-borne encephalitis virus is the only flavivirus known to circulate endemically in Sweden, and the detection of antibodies towards flaviviruses would, therefore, indicate past exposure to TBEV. The sample was also sent for a confirmatory virus neutralization test (ANSES, France), resulting in a titre of 1:160 for TBEV.

### Chromogenic In-situ hybridization for TBEV

**Fig. 6:**
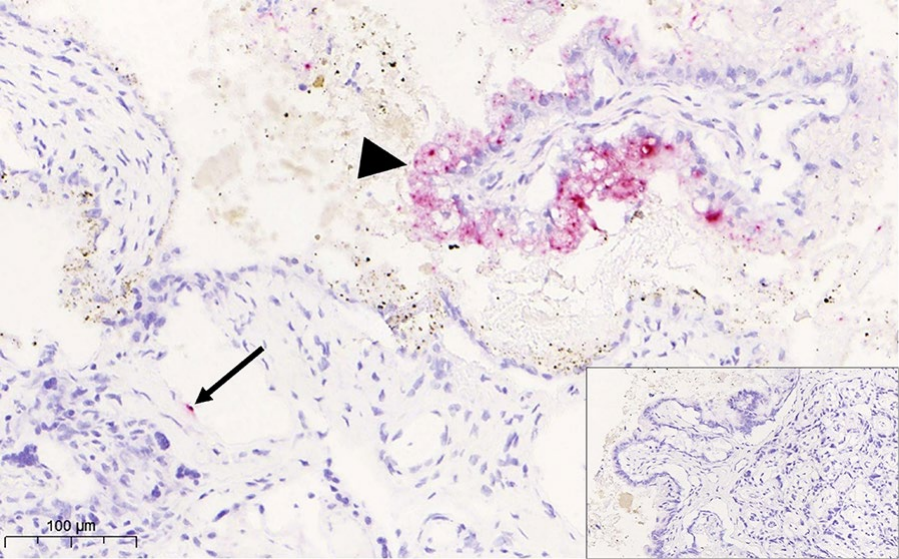
Canine placenta: viral RNA is detected also in the trophoblasts (arrowhead) of the placental junctional zone as well as in single endothelial cells (arrow). Inset: negative ISH control section processed without probe. RNA Scope ISH for TBEV

To verify the results of the viral metagenomic study and PCR results at the tissue level and investigate the localization of TBEV within the placenta, sections of placental tissue were analyzed by in situ hybridization using the RNAScope^®^ method (Bio-Techne, Advanced Cell Diagnostics, Dublin, Ireland) and the commercially available TBEV probe (V-TBEV-NS3). A brain section from a dog succumbing to TBE, confirmed by PCR of the CNS, viral sequencing, seropositivity and positive brain ISH, was used as a positive control section. A serial section, processed without the probe, was included as a negative control. There was a multifocal positive signal for TBEV in the placental sample (Figs. 5 and 6). Staining with HE of a serial section of the placenta revealed that the signal was located to the junctional zone trophoblasts with single positive cells with necrotic areas deeper in the lamellae of the labyrinth zone.

### Follow up

A telephone follow-up was performed two years later. The owner described that the bitch had regularly been treated with an acaricid, fipronil, but still repeatedly was infested with ticks, even before the death of her foetuses. The bitch had been mated with another male dog 1½ year after the caesarean section and delivered five healthy pups. The general condition of the bitch continued to be good, with no further signs of disease.

## Discussion and conclusions

We describe, for the first time, a case of vertical transmission of TBEV in a dog, associated with foetal death in late pregnancy. TBEV is the only endemic flavivirus in Sweden, and has not been associated with prenatal deaths previously. Another flavivirus, JEV, causes prenatal deaths in pigs. JEV can give litters with mummified, stillborn, and healthy offspring, as well as offspring with neurological signs [17, 31]. A similarity between the described case and JEV-infection in pigs is that all foetuses are not affected equally. This is a consequence of a vertical transmission of viruses, in contrast to when an abortion occurs due to systemic infection of the female. Transplacental infection of JEV and foetal deaths have also been described in mice [32, 33]. Vertical transmission of TBEV has been described in red voles [34] but there is no case of vertical transmission in women. There was no transfer of TBEV to the foetus in two cases of severe TBEV infection in women [35].

The aetiology of abortions and stillbirth in dogs is seldom identified, and the role of TBEV thus not known. Even if vertical transmission of TBEV leading to reproductive disturbances occurs, as suggested by the present case, it is possible that in areas where TBEV is endemic, most dogs would be exposed and immune before their first pregnancy. This is often the case with JEV for which the reproductive losses may not be as pronounced in endemic areas as in epidemic regions, since the gilts would be immune before mating [36]. The flavivirus most well-known for vertical transmission is Zika virus, after the outbreak in Brazil 2014-15, where intrauterine infections followed by microcephaly was a common manifestation [37]. Apart from Zika virus, there is scant data on vertical transmission in humans for most zoonotic flaviviruses [15]. In countries where flaviviruses circulate, many cases of abortion and stillbirth may not get an aetiological diagnosis. Foetal mortality may be high for many reasons, and the endemicity may also lead to immunity in women of child-bearing age, making it unlikely that cases are found [38]. In Japan, where JEV occurs in seasonal epidemic patterns, it has been suggested that the virus may cause seasonal infertility in women [39].

The main transmission route for TBEV is via ticks. *Ixodes ricinus* is an endemic tick in Sweden that increases in abundance and expands geographically [40], and infections with TBEV in humans are also increasing, with regional differences [41]. The seroprevalence of TBEV in dogs in Europe varies between 3 and 20% in endemic areas [18, 42, 43]. The bitch had been infested with several ticks, and a tick bite is thus the most likely route of transmission to the bitch in the present case, with following intrauterine transmission to the pups.

The bitch in the present study had PU/PD during a period from approximately day 32 to day 49 of pregnancy. It is not known if the PU/PD was related to the infection with TBEV. In humans with TBE without CNS involvement, PU/PD is usually not a reported symptom [44], neither has PU/PD been associated with TBEV infection in dogs [19]. The bitch also had a short period of inappetence, which has been described in dogs infected with TBEV [19]. Inappetence was present late in gestation, when three foetuses had already died, and there was thus no clear association with the infection. The present case suggests that TBEV infection without clinical signs in the bitch may be lethal to the developing foetuses.

Chromogenic in-situ hybridization confirmed TBEV in the placental trophoblasts and deeper labyrinthic tissue, a finding not previously reported in the dog and indicative of placental viral infection. Focal necrotic lesions of the placenta are common findings in normal, healthy pups [45]. Multifocal acute placental lamellar necrosis and microthrombosis with mild acute neutrophilia, as was seen in the present case, was associated with a higher risk of death of pups within 7 days of birth [46] but are incidental findings also in litters of healthy pups [45, 46]. Therefore, the lesions do not verify TBEV as the cause for foetal resorption and neonatal death in this case, but they raise the possibility of TBEV being a possible cause behind negative outcome in pups.

Brucellosis in dogs is difficult to rule out, since the diagnostic sensitivity of both bacterial culture and PCR are dependent on the timing of sampling, the matrix used for analysis, the handling of the sample and the methods chosen for analysis [8]. However, placenta, foetal tissues and vaginal secretions contain a high number of bacteria, resulting in abundant growth of *B. canis* when samples from infected animals are cultured on selective media [47]. The microbiological samples from the bitch and the aborted pup were readily transported to the Swedish Veterinary Agency where the culturing was performed according to standardized protocols. The process for sampling, handling samples, transport and performing the culture was thus optimal. The negative culture result, few bacteria present on histopathology, lack of lesions in the foetus and the placental tissue lesions not typical for brucellosis [48] thus make brucellosis an unlikely diagnosis, together with negative serology at follow-up. The various results on the serological tests initially performed highlight the complexity of diagnosing *B. canis* in dogs. Without available culture opportunities, and if no metagenomics had been performed, this bitch might have been falsely diagnosed with *B. canis*. Since *B. canis* is a zoonotic agent, and considered non-treatable in dogs, incorrect diagnosis of this infection might have severe consequences for dogs, owners and veterinary health personnel.

Improved knowledge about the aetiological agents behind foetal losses in dogs is important. Flaviviruses, including TBEV, may play a role as causative agents in dogs as they do in other species. This may cause increasing concerns giving climate change and the spread of vectors to new areas. In conclusion, a bitch was presented with foetal death in late pregnancy and TBEV was detected from the placental tissue of one of her foetuses, giving evidence for vertical transmission. With the spread of both ticks and TBEV, these findings indicate that further investigations of the association between TBEV and foetal death in dogs are warranted.

## Data Availability

The datasets generated and/or analysed during the current study are available in the Sequence Read Archive (SRA) repository, NCBI, under the BioProject ID PRJNA1267069, [https://www.ncbi.nlm.nih.gov/bioproject/?term=PRJNA1267069] https://www.ncbi.nlm.nih.gov/bioproject/?term=PRJNA1267069.
